# Mr. Edwards's Case of Hydropthalmia

**Published:** 1810-02

**Authors:** G. F. Edwards

**Affiliations:** Member of the Royal College of Surgeons, London. Bath


### *To the Editors of the Medical and Physical Journal.*

GENTLEMEN,

MR. ———, on a visit to this city, received an accidental blow upon the globe of his right eye, which caused him very considerable pain, accompanied with a sense of fulness in that organ; he informed me, that the sense of fulness in the eye-ball gradually increased every day, and that the power of vision diminished as the sense of fulness increased.

On examination, a very considerable quantity of extravasated blood presented itself, effused between the tunica conjunctiva and sclerotica, particularly next the external canthus, occasioned, no doubt, by the blow lacerating some of the vessels, if I may so express myself. I was struck with a beautiful congeries of arteries anastomosing round the ligamentum annulare, sent from the adnata and sclerotica; this inflammation was excited by the violence of the blow. The most particular appearance was the enlargement of the eye; the formation of a tumour by the protrusion of the transparent cornea, caused by an increased secretion of the aqueous humour. The motion of the eye-lid was much impeded, from the circumstance of the distended state of the eye. The above confirmed my first opinion, that this was a clear case of hydropthalmia; and as I considered the sight only to be impaired by the morbid distension, I proposed the operation for the hydrops occuli, before the disease was aggravated so as to burst through the cornea, and totally destroy vision. After fairly stating both sides of the question to my patient, he readily consented to undergo the operation when I conceived it prudent; consequently, the 23d of November was proposed, which was eight days from the time he met with the accident. In cases of hydropthalmia, where there is a probability of preserving vision, I prefer operating with a trocar behind the iris; but when it is to be done merely for lessening the globe of the eye, by discharging the aqueous humour, the knife is, in my opinion, to be preferred, as equally efficacious, and less painful. The head being secured, with my right thumb and finger I steadied the eye, and with a flat lancet-pointed trocar in my left hand, I made an opening in the most convenient part of the tumour, behind the iris; when the end of the canula was sufficiently covered by the tunica sclerotica, I with-

 drew drew the stilette, and suffered as much of the aqueous humour to escape as was considered necessary.

The operation being completed, my patient was put to bed, laid on his back; the part first being dressed with a compress of linen, moistened with a solution of extract Saturni, and retained by a proper bandage. At night gtt. xl. of tinct. opii were given, in order to diminish pain; a strict antiphlogistic regimen enjoined, with a low diet.

24th. Night passed tolerable easy; removed the bandage, &c.; found inflammation had succeeded to the operation, but in no considerable degree; the same plan was continued.

25th. A restless night; felt darting pain through the globe, with a sensation of dust or other extraneous matter in the eye. I removed the dressing, and found very considerable inflammation, with large distended blood-vessels running in strait lines towards the cornea, particularly on that side next the operation; the largest of these I divided with scissars. After raising them with forceps, applied the same dressings; gave a brisk cathartic, and ordered two leeches to the temple, on the side of the diseased eye.

26th. Expressed himself considerably relieved; had enjoyed a good night, and felt quite comfortable; on removing the bandage and the compress, my patient immediately exclaimed, I can see very well comparatively; the inflammation had in great part subsided; the pupil was distinctly seen, which visibly contracted on the application of light. The same plan, with little variation, was continued for some time; and when the inflammation had entirely disappeared, and absorption of the extravasated blood effected, this gentleman saw nearly as well as ever at the end of three weeks from the operation; and now no defect is visible, except a small mark where the instrument entered.

### Remarks.

This case of hydropthalmia was no doubt caused by the violence of the blow, as the disease manifested itself very shortly afterwards, denoted by the symptoms enumerated.

The extravasation of blood was from the laceration of vessels, and the natural action of the eye-lid was impeded by the over-distension of the globe. If this disease had continued, I am of opinion, it would soon have ruptured the transparent cornea, and thereby entirely destroyed the eye.

The only chance of saving the organ was by the operation, and that chance was greater, as there was no previous vious disease, and the morbid action then present was so recent.

The operation succeeded beyond expectation, by first discharging the increased contents of the eye; and 2dly, by diminishing the inflammation; and by proper tonic applications, the natural or healthy action was restored, and the functions of the eye happily completed.

G. F. EDWARDS,
Member of the Royal College of Surgeons, London.

*Bath, Jan.* 3, 1810.

